# Incidental giant renal oncocytoma: a case report

**DOI:** 10.1186/1752-1947-4-358

**Published:** 2010-11-08

**Authors:** Anastasios Anastasiadis, Georgios Dimitriadis, Dimitrios Radopoulos

**Affiliations:** 1 13 Kafantari Street, 55132 Thessaloniki, Greece; 2 Gennimatas Hospital, 41 Ethnikis Aminis Street, Thessaloniki, Greece

## Abstract

**Introduction:**

Large renal oncocytomas are not very rare entities. To the best of our knowledge, we report one of the largest oncocytomas in the English literature. The tumor was incidentally diagnosed and, based on the preoperative clinical and radiographic findings, was therefore considered to be a renal cell carcinoma.

**Case presentation:**

A 48-year-old Caucasian diabetic man had an abdominal ultrasound for chronic abdominal discomfort, which revealed a large mass on the left kidney. An abdominal computed tomography scan revealed a contrast enhancing, well defined, heterogenous large mass (16.5 × 13.9 cm) originating from the left lower pole with cystic and solid areas. A magnetic resonance imaging scan was performed with no evidence of renal vein or caval thrombus or embolus. A radical nephrectomy was performed through a left flank intercostal incision and the pathology diagnosed renal oncocytoma. The postoperative course was uneventful and the patient was discharged six days later.

**Conclusion:**

Several reports have characterised this essentially benign renal histiotype, which represents 5% to 7% of all solid renal masses. Unfortunately, most renal oncocytomas cannot be differentiated from malignant renal cell carcinomas by clinical or radiographic criteria. Central stellate scar and a spoke-wheel pattern of feeding arteries are unreliable diagnostic signs and are of poor predictive value. These tumors are treated operatively with radical or partial nephrectomy or thermal ablation, depending on the clinical circumstances. We report on, to the best of our knowledge, the fourth largest lesion of this type of renal pathology.

## Introduction

Despite the fact that oncocytomas tend to be relatively smaller and asymptomatic than renal cell carcinomas (RCCs), they cannot be reliably distinguished preoperatively. The variable nature of their presentation and the overlap of radiographic characteristics between these lesions complicate their clinical differentiation [1]. This case report illustrates the difficulty in the preoperative diagnosis of even very large, contrast-enhancing renal masses and underscores the inclusion of renal oncocytoma in the differential diagnosis of these lesions.

## Case presentation

A 48-year-old diabetic Caucasian man had an ultrasound of the abdomen for chronic abdominal discomfort, which revealed a large mass of the left kidney. There was no flank pain or any other relevant clinical symptoms. His previous personal and family history was noncontributory. At physical examination a firm mass was palpated in left upper abdominal quadrant.

Blood tests, including renal and liver function, were normal except for glucose; urine analysis and chest X-ray were also normal. Computed tomography revealed an enhancing well-defined heterogeneous large mass of 16.5 × 13.9 cm originating from the lower pole of the left kidney, with cystic and solid areas within the mass. A magnetic resonance imaging scan was performed in order to further evaluate the renal artery and vein, which showed no evidence of renal vein or caval thrombus or embolus. Due to the possibility of renal malignancy, radical nephrectomy was performed through a left flank intercostal incision.

**Figure 1:**
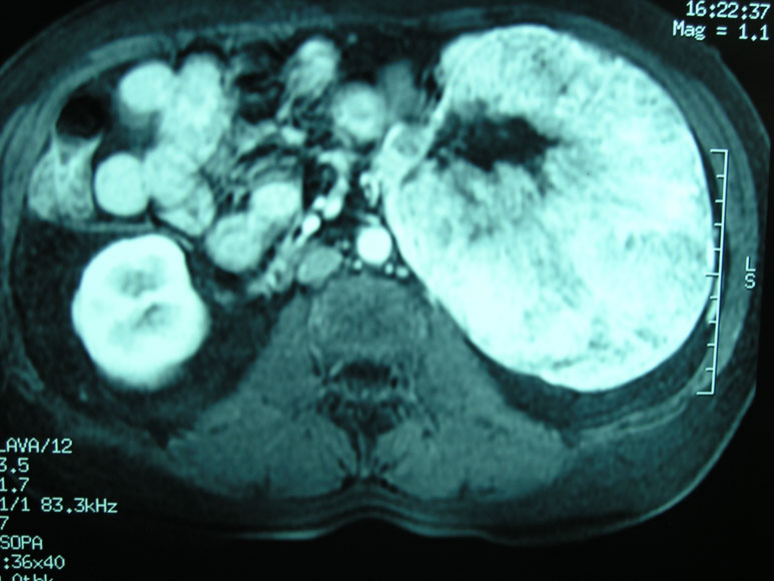
**Preoperative magnetic resonance imaging (MRI) scan**. T2-weighted sagittal MRI.

There were no postoperative complications and the patient was discharged six days after the operation. The specimen weighed 1973 g and the dimensions were 27 × 16 × 13 cm. Histopathology diagnosed a renal oncocytoma. No islets of renal cell carcinoma and no evidence of necrosis or bleeding were found. No vascular or capsular invasion was detected. The maximal diameter of the tumor was 16 cm. Immunohistochemistry was positive for epithelial membrane antigen and parvalbumin and negative for vimentin, CK7 and CD10, which further supported the initial diagnosis (Figure 1).

## Discussion

Oncocytoma is the second most common solid tumor of the kidney after RCC. They both originate from distal tubules and histologic similarities do exist, particularly for the esinophilic variant of the chromophobic carcinoma. To date, Demos *et al*. [2] have reported the largest and heaviest oncocytoma, which measured 27 × 20 × 15 cm and weighed 4652 g. Banks *et al*. [3] reported the second heaviest renal oncocytoma (3090 g, 21 × 18 × 15 cm) and Kilic *et al*. [4] reported the third heaviest oncocytoma (2680 g, 20 × 15 × 10 cm). Unfortunately, most renal oncocytomas cannot be differentiated from malignant RCC by clinical or radiographic criteria. Common imaging findings are central stellate scar and spoke-wheel pattern of feeding arteries but are usually unreliable for preoperative differential diagnosis [5,6]. Consequently, these tumors should be treated operatively like RCC with radical or partial nephrectomy and, alternatively, with thermal ablation, depending on the clinical circumstances. Even when very large, they are generally well encapsulated and are rarely invasive or associated with metastases [7].

Common cytogenetic findings for oncocytomas include the loss of the first and Y chromosomes, a loss of heterozygosity on chromosome 14q and rearrangement at 11q13. On the contrary, abnormalities of chromosomes 3, 7 and 17 are rarely found in association with oncocytomas. The genetic alterations observed with renal oncocytomas are thus characteristic and distinct from those described for the various subtypes of RCC. Despite their benign behavior, however, oncocytomas should be monitored closely and treated if there is evidence of rapid growth or a coexisting RCC, which occurs in 10% to 32% of reported patients [1].

## Conclusion

Several reports have characterized this essentially benign renal pathology which represents 3% to 7% of all solid renal masses. Unfortunately, most renal oncocytomas cannot be differentiated from malignant RCCs by clinical or radiographic evidence [6]. We report, to the best of our knowledge, the fourth largest lesion of this type of renal pathology.

## Abbreviations

EMA: epithelial membrane antigen; RCC: renal cell carcinoma.

## Consent

Written consent was obtained from the patient for publication of the case report and any accompanying images. A copy of the written consent is available for review by the Editor-in-Chief of the journal.

## Competing interests

The authors declare that they have no competing interests.

## Authors' contributions

AA conceived the study concept and design, was involved with patient care and drafted the manuscript and literature review. DG and RD were involved with the formation of the study concept and its design, patient care, the drafting of the manuscript and the literature review. RD and AA carried out the operation on the patient. All authors have read and approved the final version of the manuscript.
